# The action of Arabidopsis DICER-LIKE 2 in plant growth inhibition

**DOI:** 10.1093/plcell/koaf206

**Published:** 2025-08-29

**Authors:** Yuelin Liu, Wei Yan, Qianyan Linghu, Huijuan Tan, Hongwei Guo

**Affiliations:** New Cornerstone Science Laboratory, Shenzhen Key Laboratory of Plant Genetic Engineering and Molecular Design, Institute of Plant and Food Science, Department of Biology, School of Life Sciences, Southern University of Science and Technology (SUSTech), Shenzhen, Guangdong 518055, China; New Cornerstone Science Laboratory, Shenzhen Key Laboratory of Plant Genetic Engineering and Molecular Design, Institute of Plant and Food Science, Department of Biology, School of Life Sciences, Southern University of Science and Technology (SUSTech), Shenzhen, Guangdong 518055, China; New Cornerstone Science Laboratory, Shenzhen Key Laboratory of Plant Genetic Engineering and Molecular Design, Institute of Plant and Food Science, Department of Biology, School of Life Sciences, Southern University of Science and Technology (SUSTech), Shenzhen, Guangdong 518055, China; New Cornerstone Science Laboratory, Shenzhen Key Laboratory of Plant Genetic Engineering and Molecular Design, Institute of Plant and Food Science, Department of Biology, School of Life Sciences, Southern University of Science and Technology (SUSTech), Shenzhen, Guangdong 518055, China; New Cornerstone Science Laboratory, Shenzhen Key Laboratory of Plant Genetic Engineering and Molecular Design, Institute of Plant and Food Science, Department of Biology, School of Life Sciences, Southern University of Science and Technology (SUSTech), Shenzhen, Guangdong 518055, China; Shenzhen Branch, Guangdong Laboratory for Lingnan Modern Agriculture, Agricultural Genomics Institute at Shenzhen, Chinese Academy of Agricultural Sciences, Shenzhen 518120, China

Dear Editor,

Small RNAs (sRNAs) are crucial regulators of plant gene expression, with sRNAs of different lengths modulating gene expression through distinct mechanisms (Zhan and Meyers 2023; Vaucheret and Voinnet 2024). The Arabidopsis genome encodes 4 DICER-LIKE (DCL) endoribonucleases, of which DCL1 produces 21-nt microRNA, whereas DCL2, DCL3, DCL4 produce 22-nt, 24-nt, and 21-nt siRNA, respectively (Bologna and Voinnet 2014). Although the functions and action modes of DCL1, DCL3, and DCL4 have been extensively investigated, research on DCL2 has been relatively limited, partly due to the lack of obvious phenotypes in function-deficient mutants. Bouché et al. (2006) found that the simultaneous absence of *DCL1* and *DCL4* resulted in severe developmental defects in plants, which could be rescued by mutation in DCL2, suggesting that DCL2 activity can interfere with plant development. Despite the lack of gene complementation experiments due to the difficulty of transgenic expression, DCL2-mediated plant developmental defects in the absence of DCL4 are believed to be linked to the production of 22-nt siRNAs (Wu et al. 2017, 2020).

Recently, Nielsen et al. (2024) argued that it is the DCL2 protein, not the 22-nt siRNA, that can activate an autoimmune response, causing growth defects in plants and conferring basal antiviral resistance (Nielsen et al. 2024). This article has generated considerable interest (Doll 2024). They found that a subset of *dcl4* single mutants exhibited DCL2-dependent growth-defect phenotypes. Additionally, having DCL2 in a heterozygous state (*dcl4 dcl2-1/+*) reduced the proportion of plants showing growth defects compared to *dcl4* mutants with 2 copies of *DCL2*. However, small RNA sequencing (sRNA-seq) results revealed that *dcl4 dcl2-1/+* mutants produce 22-nt siRNAs in amounts comparable to those in *dcl4* mutants. Based on these observations, they proposed that the phenotypic defects of *dcl4* depend on the dosage of DCL2 protein but not on siRNA levels. Furthermore, by manipulating DCL2 protein amount through transgenic experiments, they found a positive correlation between the occurrence of DCL2-dependent growth defects and the expression levels of DCL2 protein, which further supports their hypothesis (Nielsen et al. 2024). Although we are appreciative of these intriguing findings, we are concerned about the interpretation of some of the genetic and sequencing data, especially the evaluation of the role of DCL2 and 22-nt siRNAs through calculating the occurrence of growth-defect phenotypes of *dcl4* mutants.

## The amounts and species of 22-nt ct-siRNAs vary in *dcl4-*harboring mutants that display distinct phenotypes

**Figure 1.**
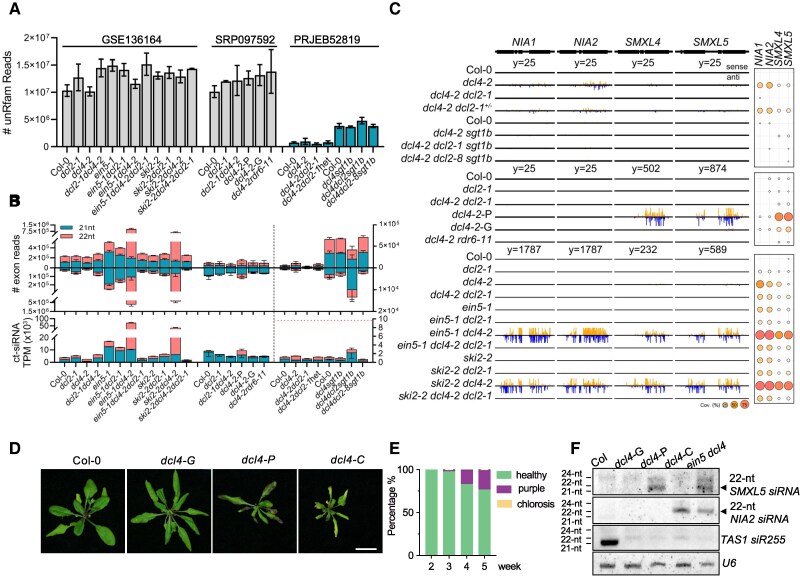
The dynamic changes of ct-siRNAs abundance and sources contribute to the phenotypes of *dcl4* mutant. **A)** Statistics of sRNA reads from sRNA-seq data from GSE136164 (136 samples, Wu et al. 2020), PRJEB52819 (65 samples, Nielsen et al. 2024), and SRP097592 (24 samples, Wu et al. 2017), respectively. Reads that aligned to the rRNA sequences from Rfam database were excluded during analysis. **B)** Statistics of sense and antisense strand-derived 21-nt and 22-nt sRNAs that aligned to exons in the indicated genotypes. The upper panel shows the read counts, and the lower panel shows the normalized TPM (tags per million). Samples from GSE136164 and SRP097592 were plotted on the left *y* axis, while samples from PRJEB52819 were plotted on the right *y* axis. The horizontal dashed line indicated the average 21-22 nt siRNA TPM in *dcl4-2*-P. **C)** Distribution of 22-nt ct-siRNAs generated from *NIA1*, *NIA2*, *SMXL4*, and *SMXL5* in indicated genotypes. Only the first replicate was shown for all the samples. The read numbers were normalized by the average numbers of sequencing within each dataset. The coverage (Cov.) percentages of small RNA reads mapped to each locus are summarized in the adjacent dot plot, where the size and color of dots correspond to the percentage coverage. **D)** Rosette morphology of 4-wk-old plants of Col-0, healthy green *dcl4* (*dcl4-G*), purple *dcl4* (*dcl4-P*), and chlorotic *dcl4* (*dcl4-C*). Scale bar = 2 cm. **E)** The proportion of developmental defects in *dcl4* changes over time. Among 225 plants examined, chlorosis was observed as a rare case under the cultivation conditions used, with a proportion of 0.44%. **F)** RNA blot detection of small RNAs derived from *SMXL5*, *NIA2*, and *TAS1* in the indicated genotypes. The analysis was performed by stripping and reprobing the same membrane. U6 spliceosomal RNA served as the loading control. The size of siRNA derived from *SMXL5* and *NIA2* based on the size of *TAS1 siR255* on the same membrane. See Supplementary Methods for experimental details.

We agree that the dosage of DCL2 might affect the probability of growth-defect phenotypes of *dcl4* mutants, as they provided evidence through mass spectrometry analysis of DCL2 protein in mutants and transgenic plants. However, we are concerned with other conclusions in this study. Sequencing results of *dcl4-2* and *dcl4-2 dcl2/+* asymptomatic plants show that the heterozygous state of DCL2 does not affect 22-nt siRNAs originating from *TAS1B*, *IR71*, *SMXL4/5*, and *NIA1/2* (Nielsen et al. 2024). By re-analyzing and comparing the sRNA-seq data from Nielsen et al. (2024) and Wu et al. (2017, 2020), we noticed a particularly low read count in the sequencing data for *dcl4-2* and *dcl4-2 dcl2/+* in Nielsen et al. (2024) (Fig. 1, A to C). Furthermore, when mapped to exons, the siRNA values in Nielsen et al. (2024) remained notably low (Fig. 1, B and C). Therefore, a more comprehensive sequencing dataset may be required to draw definitive conclusions here. Besides, the proportion of phenotype changes caused by the dosage of DCL2 protein can be explained by other mechanisms. For example, the higher the amount of DCL2 protein, the more likely it is to access dsRNA substrates to produce 22-nt siRNAs, thereby leading to more plants exhibiting growth inhibition phenotypes.

Additionally, under normal growth conditions, *dcl4* mutant plants show distinctive phenotypes: the majority are green with no obvious growth defects (*dcl4*-*G*), while the rest display 2 types of leaf-appearance defects: purple pigmentation (*dcl4-P*) or chlorosis (*dcl4-C*) (Wu et al. 2017; Nielsen et al. 2024) (Fig. 1, D and E). This observation suggests that there may be different underlying causes that result in the distinct phenotypes of the *dcl4* mutant. Accordingly, the data analysis of Wu et al. (2017) reveals different amounts of 22-nt ct-siRNAs across the *dcl4* mutant plants, indicating that *dcl4-P* plants produce higher levels of 22-nt ct-siRNAs than *dcl4*-*G* (Fig. 1C). Therefore, we question the value of only analyzing the siRNA in asymptomatic plants without comparing the siRNA profiles in *dcl4* and *dcl4 dcl2/+* mutants that exhibit different phenotypes and severities of growth defects. To adequately evaluate the role of 22-nt ct-siRNAs in DCL2-dependent plant growth defects, a comprehensive and detailed analysis of the siRNA changes in these mutants is essential. For example, if healthy-looking *dcl4-2 dcl2-1/+* generate comparable levels of ct-siRNAs to those in *dcl4* showing evident growth defects, this would better support the hypothesis that 22-nt siRNA are not involved in DCL2-mediated growth inhibition.

Nielsen et al. (2024) also generated the *stg1b dcl4-2* double mutant, which exhibited consistent DCL2-dependent developmental defects. Owing to the fertility of this mutant, the authors identified several point mutations in DCL2 that could recover the phenotype of *stg1b dcl4-2* through forward genetic screening. They argue that these point mutations in DCL2 can partially uncouple the production of 22-nt siRNAs from growth defects. According to their results, although there is still an accumulation of DCL2-generated siRNA in plants with certain DCL2 point mutations, the amount of an even larger group of siRNA production is reduced in the selected *dcl2* mutant allele. Therefore, the genetic study does not adequately support the claim of uncoupling DCL2-dependent siRNA production from growth inhibition. Additionally, according to the descriptions in their methodology, it took 4 to 6 wks for all *stg1b dcl4* individuals to display growth symptoms, yet Nielsen et al. (2024) performed their analysis on 3-wk-old seedlings, when the ct-siRNAs, particularly those originating from *SMXL4/5* and *NIA1/2*, are undetectable in their mutants (Fig. 1, B and C). This raises a possibility that the 3-wk-old plants collected for sRNA-seq may not have begun to develop growth-defect phenotypes, similar to our observation that the probability of *dcl4* mutants displaying phenotypes also increases over time (Fig. 1E). Additionally, in *ein5 dcl4* (*ed*) and *ski2 dcl4* (*sd*) mutants, where specific RNA decay factors are absent along with DCL4, severe growth inhibition phenotypes are exhibited, and large amounts of 22-nt ct-siRNAs accumulate. We found that even in these 2 mutants, the accumulation of 22-nt ct-siRNAs as well as the severity of growth defects are relatively minor at the seedling stage, becoming progressively pronounced as plants age (Feng et al. 2024). Therefore, in our view, the results of the genetic study by Nielsen et al. (2024) do not conclusively determine whether or not DCL2-dependent growth defects are coupled with 22-nt siRNAs.

## Phenotypic variation of *dcl4*-harboring mutants depends on 22-nt siRNA species

Nielsen et al. (2023, 2024) proposed an autoimmune pathway that relies on DCL2 independently of RNAi. However, this theory is difficult to explain the various types and severities of growth defects of *dcl4* as well as *ed* and *sd.* According to the current model, runaway transitive post-transcriptional gene silencing (PTGS) mediated by 22-nt siRNAs is responsible for abnormal plant development. Therefore, different growth defects of *dcl4*, such as purple and chlorotic plants, should theoretically accumulate siRNAs from distinct genes. Indeed, our RNA blot results indicate that chlorotic *dcl4*-2 (*dcl4-C*) generates siRNAs derived from *NIA1*/*2*, which are hardly detectable in *dcl4-P* (Fig. 1, D and F). This finding is consistent with the chlorotic phenotype caused by the silencing of *NIA1/2* (Elmayan et al. 1998; Bazin et al. 2023), a critical phenomenon overlooked by Nielsen et al. (2023, 2024). Additionally, our data (Fig. 1, D and F), as well as results from Wu et al. (2017), demonstrate that *dcl4-P* mainly produces siRNAs derived from *SMXL4/5* rather than *NIA1/2*, leading to purple leaf pigmentation. These results reveal that the genes generating 22-nt siRNA differ among different phenotypes of *dcl4*, and that the corresponding siRNA target gene mutants or silenced lines exhibit matching phenotypes. This suggests that the observed growth defects may be caused by the silencing of specific gene loci targeted by 22-nt ct-siRNAs in *dcl4* plants. This raises another intriguing question: what triggers the production of ct-siRNA, and how are specific coding transcripts selectively generating siRNAs in *dcl4*?

Although both *ed* and *sd* exhibit severe developmental defects, there are still phenotypic differences between them; *sd* presents needlelike leaves, whereas *ed* does not (Wu et al. 2020). By analyzing the cognate genes of ct-siRNA in *ed* and *sd*, we found that *sd* specifically accumulates high levels of siRNAs derived from *PHA*, *PHB*, *REV*, and *HB-8*, which are associated with leaf polarity (Prigge et al. 2005; Zhou et al. 2007; Wu et al. 2020). The silencing of these genes may be the reason for the needlelike leaves observed in *sd*. Therefore, we believe that the gene silencing by 22-nt siRNA provides a better explanation for the *sd*-specific phenotype than the induction of autoimmunity.

We believe the weight of evidence thus far suggests that the variety of growth defects dependent on DCL2 results from silencing of different target genes by 22-nt siRNAs. Conversely, Nielsen et al. (2023) proposed that the plant growth defects are caused by DCL2 activating RNAi-independent autoimmunity during its catalytic turnover, based on the evidence that mutations in 2 R genes, including the plasma membrane-localized CNL encoded by *L5* and the TNL *RPP9/RAC1*, can restore the proportions of *dcl4* and *stg1b dcl4* showing growth defects. Nevertheless, this genetic evidence does not exclude an indirect effect, as their model would require DCL2 to either activate different R proteins or trigger varied signaling pathways through the same R protein to explain the distinct phenotypes of *dcl4-*harboring mutants. This hypothesis lacks concrete empirical support. Moreover, it is not surprising that immune signaling is activated in growth-defective *dcl4* mutants that accumulate 22-nt ct-siRNAs. mRNA-seq results from *ed* and *sd* show significant activation of defense signals, such as jasmonic acid and salicylic acid, suggesting that plants may activate immune responses directly or indirectly via DCL2/22-nt siRNA (Wu et al. 2020). Numerous immuno-activated mutants show severe growth inhibition phenotypes (Rivas-San Vicente and Plasencia 2011; Huot et al. 2014; Van Butselaar and Van Den Ackerveken 2020). Additionally, some RNA metabolism mutants, such as *upf1*, *smg7*, *dhh1-like*, and *dxo1* exhibit growth defects accompanied by activation of immune signals, and their phenotypes can be partially recovered by some immune mutants (Riehs-Kearnan et al. 2012; Chantarachot et al. 2020; Pan et al. 2020). Thus, the genetic restoration of the *dcl4* growth defects by certain R gene mutations does not necessarily indicate that DCL2 protein activates immune response.

## Multiple PTGS factors are required for DCL2-dependent growth inhibition

In the plant PTGS pathway, many protein factors are involved and play their specific functions (Zhan and Meyers 2023). Among them, Argonaute 1 (AGO1) is required for the action of sRNA. As shown by Nielsen et al. (2024), in *dcl4-2* and *dcl4-2 dcl2-1/+*, 22-nt siRNAs derived from *SMXL4/5* and *NIA1/2* are loaded into AGO1. However, whether these RNA-induced silencing complexes (RISCs) are functional, and whether the defects in AGO1 function can recover the growth restriction of *dcl4-2* and *dcl4-2 dcl2-1/+−*, has not been shown. In contrast, Wu et al. (2020) found that 2 mutant alleles of AGO1 could recover the growth defects of *ed* and *sd*, indicating the important role of DCL2-dependent 22-nt siRNA-RISC in growth defects (Wu et al. 2020). Plant sRNAs can be methylated by HUA ENHANCER 1 (HEN1) to prevent their 3′ end uridylation and degradation by nucleases (Yu et al. 2005; Ren et al. 2012; Zhao et al. 2012). *hen1-8*, a weak allele of the *HEN1* mutant, partially but significantly recovered the severe growth defects of *ed* and *sd* (Wu et al. 2020). Since the defect in HEN1 function likely affects the maturation and stability of sRNAs but does not affect the catalytic activity of DCL2 protein, the genetic evidence presented by Wu et al. (2020) demonstrates that 22-nt siRNAs, but not DCL2 protein per se, cause plant growth defects. To ascertain an RNAi-independent role of DCL2 proposed by Nielsen et al. (2024), it should be tested and shown whether mutations in *AGO1* and *HEN1* can recover the phenotype of *stg1b dcl4*.

## Conclusion

In summary, we acknowledge that Nielsen et al. (2024) designed numerous sophisticated experiments to demonstrate that DCL2-dependent phenotypes are independent of RNAi. We also agree the dosage of DCL2 influences the proportion of growth defects in *dcl4* mutants. However, we disagree with the hypothesis that the DCL2-dependent growth inhibition relies on the DCL2 protein level rather than on the 22-nt siRNA-mediated gene silencing, as current evidence appears insufficient to support this claim. (i) Nielsen et al.'s sequencing data are insufficient to reliably prove that the heterozygous state of DCL2 does not affect siRNA production. (ii) The levels and species of 22-nt ct-siRNAs are variable, and this study did not thoroughly analyze the siRNA in *dcl4* and *dcl4 dcl2/+* showing different phenotypes; instead, the analysis was likely biased toward asymptomatic plants. (iii) Nielsen et al. (2024) do not explain what factors lead to *dcl4-2* exhibiting distinct growth defects, however, the evidence provided by Wu et al. (2017, 2020) demonstrates the consistency between the species of ct-siRNAs and the distinct phenotype of *dcl4*-harboring mutants. (iv) The growth defects of *dcl4-*harboring mutants depending on the RNase III and Helicase activities of DCL2, as well as the requirement of AGO1 and HEN1 presented by Wu et al. (2020) suggest that the production and the function of 22-nt siRNAs are crucial for DCL2-dependent growth defects. (v) The restoration of *dcl4* and *stg1b dcl4* showing growth defects by R gene mutations could be an indirect effect. Taken together, we believe that the evidence currently provided by Nielsen et al. (2024) for their proposal that DCL2-dependent growth inhibition is uncoupled from its canonical function in 22-nt siRNA production remains insufficient. Further direct biochemical studies are needed to clarify the role of DCL2 in plant growth inhibition.

## Supplementary Material

koaf206_Supplementary_Data

## Data Availability

All data are incorporated into the article and its online supplementary material.

## Supplementary data

The following materials are available in the online version of this article.

Supplementary Methods. Used in this letter.
