# Supplementary material for: The action of Arabidopsis DICER-LIKE 2 in plant growth inhibition
Source: Plant Cell. 2025 Aug 29;37(8):koaf206. doi: 10.1093/plcell/koaf206 (PMC12396362; doi:10.1093/plcell/koaf206)
Supplement: koaf206_Supplementary_Data [file koaf206_supplementary_data.docx]

**Methods**

**Small RNA northern blot**

Small RNA was isolated using RNAiso for Small RNA (Takara, # 9753A) according to the manufacturer’s instruction. The sRNA was then separated in a denaturing 16% polyacrylamide gel and then transferred to a neutral nylon membrane (Hybond-NX, GE Healthcare, #RPN303). After UV cross-linking, the membrane underwent pre-hybridization, hybridization with Dig-labelled oligonucleotide probes followed by incubation with anti-Dig-AP (Roche, # 11093274910; 1:20000 dilution). The sRNA on membrane was visualized using Tanon 5200 Multi chemiluminescent imaging system (Tanon) after incubated with CDP-Star (Roche, # 12041677001). The sequence of the Dig labeled probes were as follows：*TAS1 siR255*， 5’-TACGCTATGTTGGACTTAGAA-3’; *NIA2*, 5’-GAGCAATACCCGGACCGGTTAA-3’; *SMXL5*, 5’- GGCGTCCAAGTTCATGAGAAGA-3’; U6, 5‘-AGGGGCCATGCTAATCTTCTC-3’.

**Small RNA-seq data analysis**

Before reads alignment, the sequencing adapter sequences were trimmed from the rawdata with TBtools using default parameters (Chen et al. 2020). Then the 18-26-nt clean reads were firstly aligned to the ribosomal RNAs (http://rfam.xfam.org/) of Arabidopsis using bowtie (Langmead et al. 2009) to remove rRNA-derived fragments, with 2 mismatches allowed (-v 2). Then the unmapped reads were aligned to the TAIR10 reference genome using bowtie without mismatch (-v 0 -M 8). Reads mapped to protein coding genes, untranslated regions (UTRs), lncRNAs, transposon elements were annotated with bedtools (Quinlan and Hall 2010) based on the TAIR10 annotations. The protein coding genes-derived siRNA were then normalized by the total aligned reads for calculating abundance (transcripts per million, TPM). The distributions of reads generating from selected genes were normalized by the total aligned reads for visualization. For calculating the coverage of 22nt ct-siRNAs on selected genes, all the loci covered by at least one reads were counted and divided by the total exon length of each gene.

References

**Chen C, Chen H, Zhang Y, Thomas HR, Frank MH, He Y, and Xia R**. TBtools: an integrative toolkit developed for interactive analyses of big biological data. Mol Plant. 2020:13(8):1194–1202. https://doi.org/10.1016/j.molp.2020.06.009

**Langmead B, Trapnell C, Pop M, and Salzberg SL**. Ultrafast and memory-efficient alignment of short DNA sequences to the human genome. Genome Biol. 2009:10(3):R25. https://doi.org/10.1186/gb-2009-10-3-r25

**Quinlan AR and Hall IM**. BEDTools: a flexible suite of utilities for comparing genomic features. Bioinformatics. 2010:26(6):841–842. https://doi.org/10.1093/bioinformatics/btq033
